# Ultrasonography helps emergency physician identify the best lumbar puncture site under the conus medullaris

**DOI:** 10.1186/s13049-017-0406-9

**Published:** 2017-06-27

**Authors:** Line Dussourd, Batistin Martinon, Clara Candille, Carole Paquier, Claire Wintenberger, Perrine Dumanoir, Anais Plazanet, Damien Viglino, Maxime Maignan

**Affiliations:** Emergency Department, CHU Grenoble Alpes, CS 10217, 38043 Grenoble, cedex 9 France

**Keywords:** Ultrasonography, Lumbar puncture, Emergency

## Abstract

**Background:**

Ultrasonography – assisted lumbar puncture helps physicians identify traditional anatomical landmarks. However, it could help to overcome the anatomical dogmas and thus identify the best interspinous space under the medullary cone.

**Methods:**

Traditional anatomical landmarks were reported on a tracing paper in patients with an indication for lumbar puncture. Then, ultrasonography was used to locate the optimal interspinous level defined as the widest subarachnoid space located below the conus medullaris. Primary endpoint was the distance between traditional and ultrasound landmarks.

**Results:**

Fifty-seven patients were included. Seven emergency physicians practiced the procedure. The median absolute distance between traditional anatomical landmarks and ultrasound marking was 32 [interquartile (IQR) 27 – 37] mm. The inter-spinous space identified in the two procedures was different in 68% of the cases.

**Conclusions:**

Ultrasound not only allows us to better identify anatomical structures before lumbar puncture, but it also allows us to choose a site of puncture different from recommendations.

Dear Editor

Lumbar puncture is a common procedure in emergency departments, with 0.8 procedures per 100 admissions [1]. The failure rate of this procedure is close to 10% and complications such as postdural puncture headache are not uncommon [2]. There is increasing evidence for the benefits of ultrasound-assisted lumbar puncture, particularly in children and infants [2, 3]. The main benefit of ultrasound localization of puncture site is the easy identification of traditional anatomical landmarks [4, 5]. In fact, anatomical assessment of intervertebral space level for lumbar puncture is misleading in more than 36% [6]. The rates of failure and complications after lumbar puncture are thus diminished due to the anatomical localization capabilities of ultrasonography [2]. However, we believe that the main interest of ultrasonography studies lies in a more comprehensive anatomical approach. Instead of simply locating the interspinous space L3-L4 or L4-L5, ultrasound allows the location of the optimal interspinous level below the conus medullaris. This approach has been recently tested in infants [3]. We have also used this approach in adults to investigate the impact of ultrasonography on the choice of the lumbar puncture site.

## Methods

Patients gave their written informed consent and the study was approved by the local ethic committee. We selected patients presenting to the emergency department with an indication for lumbar puncture because of febrile or sudden headache. Whether or not the lumbar puncture was performed was left to the discretion of the emergency physician in charge of the patient. The physician performing the study was not involved in patient care. The physician in charge of the patient was not kept informed of the results of the patient. Emergency physicians performing the procedures had different level of experience (2 residents, 1 chief resident, 3 attending physicians and 1 associate professor). They were all accustomed to using ultrasound in daily medical practice (FAST echo, basic cardiac ultrasonography etc.). Before participating in the study, they were specifically trained by an anesthesiologist to identify interspinous levels. This training did not exceed 30 min.

Briefly, the procedure for the study was performed with a Vivid S70 device (GE healtcare, Little Chalfont, UK). There was no supervision or validation of the anatomical and ultrasound procedures. Patients were randomized to have a location of the puncture site in a sitting position or in lateral decubitus to avoid any bias linked to patients’ position. Once in position, a tracing paper was placed on the back of the patient to make four marks with a surgical marking pen. This process made it possible to reposition the tracing paper in a reproducible way. An emergency physician carried out the traditional anatomical landmark of the lumbar puncture site, and reported its location only on the tracing paper. He then used ultrasound to locate the optimal interspinous level defined as the widest subarachnoid space located below the conus medullaris. The location identified by ultrasound was reported on the tracing paper. The distance between the two marks was measured as well as the distance on the carniocaudal axis and the mediolateral axis. Measurements were made by an independent emergency physician.

## Results

**Fig. 1:**
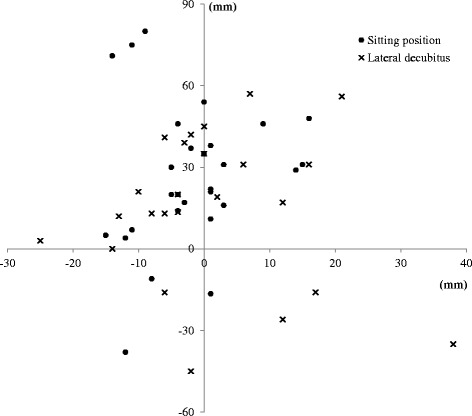
Craniocaudal and mediolateral distances between ultrasound marking and traditional anatomical marking. The center point represents ultrasound marking. Cross: sitting position; Circle: lateral decubitus

We included 52 patients including 17 (33%) men. The mean age was 45 (95% CI (18 – 84)) years and body mass index was 25 (range 17 to 41) kg/m2. Patients were admitted to the ED for meningeal syndrome (*n* = 9, 17%), sudden headache (*n* = 2, 4%), unusual headache (*n* = 27, 52%), and febrile headache (*n* = 14, 27%). 28 markings were made in sitting position and 24 in the lateral decubitus. The median absolute distance between traditional anatomical landmarks and ultrasound marking was 32 [interquartile (IQR) 27 – 37] mm. The ultrasound mark was almost systematically below the anatomical mark which corresponded to a distance on the craniocaudal axis of 29 [IQR 24 – 34] mm (Fig. 1). The distance on the mediolateral axis was 8 [IQR 6 – 10] mm. The rate of change of interspinous space was 68% in the sitting position group and 67% in the lateral decubitus group. There was no significant correlation between body mass index (BMI) and the distance between traditional anatomical landmarks and ultrasound marking (Spearman correlation coefficient: 0.006, *p* = 0.97). Obese patients with a BMI of greater than 30 kg/m^2^ tended to have a higher risk of interspinous space change (risk ratio = 1.45 [95% confidence interval: 1.06 - 1.99]; *p* = 0.14 Fisher exact test).

## Discussion

Our results confirm the value of ultrasonography to correctly identify both anatomical landmarks (midline, spinous processes etc.) and the optimal interspinous space for lumbar puncture. Ultrasound marking was mostly located below the anatomical palpation marking, which is in line with previously published data [7, 8]. Ultrasonography marking did not seek to identify the inter-spinous space L2-L3 or L3-L4, but the intervertebral space which seemed to be the most conducive to the introduction of a lumbar puncture needle. This choice was voluntary in order to be as close as possible to the realization of procedure in the emergency department. Interestingly, the conus medullaris ends in most cases in the intervertebral space L1-L2 but in 2 to 5% of cases it descends to below the vertebral body of L2 [9]. Furthermore, the shape of thecal sac varies in lower interspace making the success of the lumbar puncture more uncertain [10]. We thus believe that the main advantage of ultrasound marking is related to the identification of lower interspinous level than those traditionally chosen after anatomical marking. Our results suggest that the benefits of the ultrasound technique would be even greater in obese patients, which is consistent with previous reports [11]. Ultrasound marking allows a safe needle introduction to be made below the conus medullaris while allowing the catheterization of the thecal sac even in low interspinous spaces. Regarding the abundant literature advocating very explicitly the use of this technique, a randomized clinical trial comparing success rates using the two techniques in adults should now be carried. A study on the impact of ultrasonography on reducing the rate of post dural puncture headache could also be interesting.

## Conclusions

Ultrasound not only allows us to better identify anatomical structures before lumbar puncture, but it also allows us to choose a better site of puncture. Regarding the abundant literature advocating very explicitly the use of this technique, a randomized clinical trial comparing success rates using the two techniques in adults should be carried. A study on the impact of ultrasonography on reducing the rate of post dural puncture headache could also be useful.

## Abbreviations

IQR: Interquartile
